# Cost of Illness for Patients with Arthritis Receiving Multidisciplinary Rehabilitation Care

**DOI:** 10.1155/2011/487025

**Published:** 2011-07-26

**Authors:** Margreth Grotle, Kåre Birger Hagen, Till Uhlig, Eline Aas

**Affiliations:** ^1^Department of Rheumatology, National Resource Center for Rehabilitation in Rheumatology, Diakonhjemmet Hospital, 0319 Oslo, Norway; ^2^FORMI, Oslo University Hospital, Ullevaal, 0407 Oslo, Norway; ^3^Department of Rheumatology, Diakonhjemmet Hospital, 0319 Oslo, Norway; ^4^Department of Health Management and Health Economics, University of Oslo, 0318 Oslo, Norway; ^5^Center for Health Economic Evaluation (HEHØ), Oslo University Hospital, 0318 Oslo, Norway

## Abstract

*Purpose*. To describe healthcare consumption and costs prior to, during, and after multidisciplinary rehabilitation due to arthritis. *Methods*. 306 patients (age 18–75 years) with arthritis scheduled for multidisciplinary rehabilitation care in 9 rehabilitation centres and 4 rheumatology hospital departments were included and followed for 6 months. Costs were estimated in Euros (€) for the total sample and five clinical subgroups. *Results*. Healthcare costs ranged from €3,033 to €91,336 and were significantly higher at hospital departments compared to rehabilitation centres: €9,722 (SD 5,406) and €4,250 (SD 1,040). While total costs prior to and after rehabilitation were stable for those receiving rehabilitation at a hospital, there was a significant increase in costs for those being at a rehabilitation centre. Total mean costs were more than doubled when including social costs: from €32,410 (95% CI 20,074–37,017) to €51,491 (95% CI 49,055–61,657). *Conclusions*. Healthcare and social costs for arthritis rehabilitation were substantial both before and after a rehabilitation stay. It is important to explore methods to reduce the length of rehabilitation stay and production loss connected to rehabilitation of patients with rheumatic disease.

## 1. Introduction

Despite advances in pharmacological treatment, work disability among individuals with chronic arthritis is substantial [1], and nonpharmacological treatment and rehabilitation are necessary for many individuals. In Norway there has been a large increase in disability pension due to rheumatoid arthritis, osteoarthritis, and soft tissue rheumatism during the past 30 years [2]. Since arthritis patients contain a substantial share of patients in the healthcare sector, it is important to estimate both the use and need for healthcare services and related costs. Estimation of costs will allow more informed judgements on disease management. 

In Norway, multidisciplinary rehabilitation care for patients with inflammatory or noninflammatory arthritis is available in rheumatology departments at national, and regional hospitals and in specialised rehabilitation centres. Rheumatology hospital departments offer multidisciplinary rehabilitation care through in- and outpatient programmes, and the teams usually consist of several types of health professionals, for example, rheumatologist, nurse, physical therapist, occupational therapist, social worker, and psychologist. The teams in the rehabilitation centres are often limited to rheumatologist, nurse, and physical therapist, and hence, there is a less costly health service. There exists no unanimous practice for referring arthritis patients in need of rehabilitation care to a hospital or a rehabilitation centre. However, since patients referred to a rehabilitation centre must be able to take care of basic self care activities, it is expected that these patients have a lower level of disease severity, for example, as measured by physical function outcomes. Similarly, it is expected that patients, who can attend outpatient rehabilitation programmes at hospitals, have less activity limitations than patients in need of inpatient multidisciplinary rehabilitation care. 

A few previous studies have shown that multidisciplinary rehabilitation care, provided in outpatient rehabilitation programmes, has significantly lower costs than inpatient programmes [3–5]. In an observation study from Germany [6] the costs of inpatient rehabilitation for musculoskeletal disorders (mostly rheumatoid arthritis and back pain) also were significantly higher as compared to outpatient rehabilitation with mean cost estimates of Euro (*€*) 2047 versus *€*1111 in inpatient/outpatient, respectively. Other observational studies of inflammatory arthritis patients in hospitals report that total costs are significantly influenced by severity of the disease, such as level of pain, presence of rheumatoid factor, and grip strength [7]. Despite that there have been several costs of illness studies for patients with various arthritis diseases [6–12], few studies so far have studied the healthcare consumption and related costs among people who are in need of rehabilitation due to arthritis. 

The purpose of this study was to describe healthcare consumption and related costs over a period 3 months prior to, during, and 6 months after receiving multidisciplinary rehabilitation care for various clinical subgroups of patients with inflammatory or noninflammatory arthritis. A second aim was to investigate the effect of diagnosis, clinical setting, age, gender, severity of disease, comorbidity, and work status on total healthcare costs. We expected that patients with arthritis had a reduced need for healthcare in the first months following multidisciplinary rehabilitation care of at least one week duration. Further, we expected that the healthcare costs were higher by higher age, higher severity of disease (as measured by diagnosis, physical function outcomes, co-morbid conditions), for patients who were on sick leave, and higher among patients referred to a rehabilitation stay in a hospital as compared to a rehabilitation centre.

## 2. Methods

### 2.1. Design and Setting

 This was a multicenter, longitudinal observational study in which all institutions/hospital units, which provided multidisciplinary rehabilitation care, in South-East Norway were invited to participate. There were 15 rehabilitation centres and 4 rheumatology units in hospital eligible, of which 9 specialised rehabilitation centres and the 4 rheumatology hospital departments agreed to participate in the study. Eligible patients at the participating centres were consecutively recruited over a three-month period from September to December in 2006 and followed in 6 months after discharge.

### 2.2. Patients

All patients aged 18 to 75 years with an inflammatory joint disease or osteoarthritis of any location and scheduled for a rehabilitation stay of at least one week were invited to participate in the study. Patients diagnosis was confirmed by a rheumatologist at each of the clinical sites. Exclusion criteria were serious psychiatric comorbidity or inability to communicate in written Norwegian. The patients were recruited by research assistants at the institutions/departments, who provided information about the study to the patients, including informed agreement and written consent. The study was approved by the regional committee for medical research ethics.

### 2.3. Demographic and Health Status Data

At admission physicians examined the patients and provided diagnostic information. The patients filled in a comprehensive questionnaire at admission and discharge and a postal questionnaire at six-month follow-up. The baseline questionnaire included sociodemographic and health status variables. Sociodemographic variables concerned age, gender, level of education, work status, co-morbidity (number of diseases from a list of 12 possible diagnostic groups), use of medication (pain, inflammatory, relaxation), and use of assistive tools. Health outcomes were the Modified Health Assessment Questionnaire (MHAQ) [13] and the two component summary scores of the Short-Form 36-item (SF-36) Health Survey [14]. The SF36 Physical and Mental sum scores were derived from the eight subscales, scored from 0 to 100 where 100 is the best possible functioning. In MHAQ each question is graded from 0 to 3 where 0 is “without any effort” and 3 is “not capable”, which give an overall mean score.

### 2.4. Healthcare Consumption and Costs

Use of health care services during the 3-month period before the rehabilitation stay was collected in the baseline questionnaire before the patients started their rehabilitation. Use of health care services in the 6-month follow-up was recorded by a monthly cost diary, including registrations of number of visits to a general practitioner, physical or manual therapist, medical specialist, social worker, and alternative therapist, number of days of hospitalization and/or rehabilitation, use of medication (both on prescription and over the counter medication), and number of days of sick leave from work. Different types of healthcare costs are presented in four cost groups: cost group 1 considers costs for primary healthcare services, cost group 2 for secondary healthcare services, cost group 3 covers use of medication, and cost group 4 provides costs for production loss for employed patients. The four cost groups are presented in Table 1.

 Information on cost per unit was collected from different sources (Table 1). Costs per unit for visits to the general practitioner, specialists, and out-patient rehabilitation, which were collected from Norwegian Labour and Welfare Administration (NAV), were based on the fee for service compensation, where the copayment and fee for service on average represent 40% of the cost per consultation (Ministry of Health and Care Services, 2003).

 The rehabilitation length was calculated as number of days between admission and discharge, including the weekends (7 days per week). Costs incurred at the hospital were based on diagnosis-related groups (DRGs) 2006. DRG is an international coding system, used for administrating both clinical and financial activity, in the specialist health care. In Norway there are about 500 DRG groups. All DRG groups are given a specific weight to reflect the treatment intensity relative to the average patient. The cost for a DRG group is estimated by multiplying the cost weight for that specific DRG group with the cost of one DRG. In 2006 the cost of one DRG was *€*3,513* (Norwegian Health Directorate, 2007). When the DRG group was impossible to define, the unit cost was based on an average DRG per day.

Unit cost for the different types of treatment (physiotherapy, manual therapy, etc.) was based on charges. For the group “others” we used the lowest charge of the other treatments as an estimate. Medication costs were based on the price list from the Norwegian Medicines Agency. The costs of acupuncture, homeopathy, and other types of treatments were based on out of pocket cost reported by the patients. The cost of sick leaves (for those who were employed) is estimated to be equal to average income inclusive social costs [15]. We thus apply the human capital approach, assuming that the time one person is absent from work is lost and is therefore a cost for society [16]. The costs for medication use and sick leaves during the 6-month follow-up were estimated as an average sum per month (for all patients in the subgroups) according to the reporting in the monthly diary. All costs were transformed to Euro (*€*) with 1 *€* = 8 NOK.

In Norway, the social security system covers almost all costs related to hospital stay, including the costs for medication and other treatments during the stay, and the costs related to production loss. Furthermore, the social security system covers the main costs for rehabilitation stay and use of primary care health services for people with a rheumatic disease diagnoses, whereas there is a minor out-of-pocket payment for these services. The out-of-pocket payment for a stay at the rehabilitation institutions is in average *€*15. However, there is an upper limit for out-of-pocket costs of approximately *€*250 for Norwegian people. Exceptions are alternative treatments and treatment procedures not referred by a medical doctor, which have to be covered in total by the patient him/herself. 

### 2.5. Statistical Analysis

Continuous variables are presented by means with standard deviations and categorical variables are presented by frequencies and percentages for the total sample and for each of the diagnostic subgroups. As the cost data were highly skewed, we present the total costs with both median (with min-max) and mean with standard deviation. Cost estimates within the subgroups are presented with mean and confidence intervals, which were estimated with the bootstrap method. For differences in trends within the cost groups and between clinical subgroups, we used rank sum based on “Kruskal-Wallis equality-of-populations rank test” according to the clinical subgroups and time periods, respectively. 

When assessing associations between the total costs and potential predictors, we used the total healthcare costs in groups 1, 2, and 3 for the rehabilitation stay and the 6-months follow-up as main outcome. The effects of clinical subgroup, age, gender, comorbidity, working status, baseline scores of the MHAQ, SF36 physical and mental function, and use of assistive tool were tested separately in the inflammatory and non-inflammatory subgroups by means of Ordinary Least Square regressions. Statistical analysis was performed with SPSS 14.0 and STATA 10.5% level of significance was used.

## 3. Results

### 3.1. Sample Characteristics

Of 581 eligible patients a total of 460 (72.2%) accepted to participate in the study. There was incomplete diagnostic data for 3 patients, 23 patients withdrew before they started the rehabilitation, and 70 patients dropped out in the period between discharge from the rehabilitation stay and 6 months of follow-up. Of the 367 responders to 6-month follow-up 61 patients had lacking or incomplete data for the use of healthcare, leaving a total of 306 patients for current analyses. There were no significant differences between the initial included study sample and the final set of 306 responders with regard to diagnostic group, clinical setting, gender, work status, co-morbidity, and baseline physical function (MHAQ and SF36 Physical function sum score). The non responders, however, were slightly younger, were more frequently single, and had lower level of education and poorer mental health scores according to the SF36 Mental health.

The mean age was 61 years (SD 9.6), 75% were female, and the majority had an old age- or disability-related pension (68%) (Table 2). The majority of the patients had a primary diagnosis of osteoarthritis (123 hip osteoarthritis, 58 knee osteoarthritis, 14 polyarthritis) and approximately 36% of the patients had an inflammatory arthritis diagnosis (42 rheumatoid arthritis, 47 ankylosing spondylitis, 15 connective tissue disease, 7 polymyalgia). Nearly all the patients with osteoarthritis had rehabilitation at one of the rehabilitation centres, whereas patients with inflammatory arthritis had rehabilitation in either a hospital department (*n* = 60) or rehabilitation centre (*n* = 60). A minor subgroup of 14 patients had rehabilitation due to inflammatory arthritis in an outpatient clinic at one of the hospitals. Patients at rehabilitation centres and hospital departments differed significantly with regard to several of the sociodemographic variables at baseline: for example, patients at the rehabilitation centres were older, more likely to be female, and less likely to be employed and had more co-morbid conditions. Patients in the hospitals, however, used more inflammatory medications and also tended to use more assistive tools. There was, however, no statistical significant difference between the five subgroups with regard to use of assistive tools and use of relaxing/sleeping medication. 

The mean duration of the rehabilitation stay was significantly shorter at the hospital units (mean days 15.7, SD 5.6) than at the rehabilitation institutions (mean days 22.7, SD 5.5) (*P* < .001) (Table 2). At baseline the patients with postsurgery osteoarthritis at the rehabilitation centres had significantly worse physical function according to both MHAQ and SF36 Physical function scores as compared to the other subgroups (Table 2). The patients with an inflammatory diagnosis receiving rehabilitation in the outpatient clinic had best physical function scores and poorest SF 36 Mental score. 

### 3.2. Healthcare Consumption and Related Costs prior to, during and after the Rehabilitation Stay

Mean costs related to health consumption in the primary healthcare services (cost group 1), secondary healthcare services (cost group 2), use of medication (cost group 3), and production loss (cost group 4) for each of the five subgroups prior to, during, and after the rehabilitation stay are presented in Table 3. The total costs for the rehabilitation stay were highest at inpatient hospital departments with mean 15,723 (95% CI; 13,705–17,874), whereas the costs were lowest for the patients receiving rehabilitation at the outpatient hospital department with mean 5,120 (3,835–5,272). The mean total costs at the rehabilitation centres were 10,448 (9,883–11,013) for the inflammatory subgroup, 9,976 (8,870–10,121) for the OA conventional subgroup, and 7,770 (6,586–7,824) for the OA postsurgery subgroup. The direct costs contributed mainly to the differences in total costs between the clinical subgroups. Costs due to production loss were approximately as high as the direct cost for the rehabilitation stay and showed a similar pattern across the clinical subgroups. For all the clinical subgroups the costs related to inpatient hospital care (cost group 2) and production loss (cost group 4) accounted for the largest proportion of the total healthcare costs, whereas the costs related to primary healthcare and medication accounted for a minor part.

Healthcare consumption and costs during the 3-month period prior to and 6-month period after multidisciplinary rehabilitation care were similar with no significant changes within the two groups with inflammatory arthritis treated at a hospital. For the three clinical subgroups receiving rehabilitation at a rehabilitation centre, there was a significant increase in mean costs due to healthcare consumption in the primary care, whereas there was a significant decrease in mean costs of secondary healthcare services for the two osteoarthritis subgroups. There was also a significant decrease in use of medication in the postsurgery osteoarthritis subgroup across the time periods. Furthermore, the mean costs due to medication use were higher among patients with an inflammatory diagnosis as compared to patients with osteoarthritis within all the time periods. 

The costs related to production loss were the highest across all the clinical subgroups and were 2-3 times higher than total healthcare costs (Table 4); the total mean costs of primary care, secondary care, and medication (sum of cost groups 1, 2, and 3) were more than doubled when including the costs related to sick leave (sum of cost groups 1, 2, 3, and 4) from mean *€*32,410 (95% CI 20,074–37,017) to *€*51,491 (95% CI 49,055–61,657), respectively (Table 4). 

The arthritis-related healthcare costs (sum of cost groups 1, 2, and 3) ranged from *€*3,033 to *€*91,336 during the approximately 10 months of observation. The surgically treated osteoarthritis patients (receiving hip/knee prosthesis before rehabilitation) and the inflammatory patients at inpatient hospital departments incurred more than twice healthcare costs than the other three clinical subgroups (Table 4). When summing up all four Cost groups, including the costs due to production loss, the costs ranged from *€*5,714 to *€*134,859 during the observation period. 

### 3.3. Factors Influencing Total Costs after Rehabilitation Stay

The average total healthcare cost (sum of Cost groups 1, 2, and 3) from inclusion in the multidisciplinary team rehab stay until 6-month follow-up was mean *€*39,624 (95% CI 32,896–46,336). As MHAQ, SF36 Physical function, SF36 Mental health, and assistive tools all are variables indicating health status, we estimated five different models including different variables (Tables 5(a) and 5(b)). 

For patients with inflammatory disease all the models showed that the total costs in a hospital unit were significant more costly than rehabilitation in a hospital outpatient and at a rehabilitation centre (Table 5(a)). Being in an inpatient hospital unit implies an increase in total costs with about *€*13,000 during rehabilitation and 6 months after the rehabilitation stay. Female gender, use of assistive tools, and poor physical function in terms of either MHAQ or SF36 Physical function also had a significant effect on the healthcare costs. The correlation between the MHAQ and use of assistive tools could explain why MHAQ did not have a significant effect in Models 1 and 3. In these models, age, work status, comorbidity, and mental function did not have an effect on use of health care services among patients with inflammatory arthritis.

For the patients with osteoarthritis there was no significant difference in total costs across the other three clinical subgroups at the rehabilitation centres (Table 5(b)). Only female gender had a significant effect on the costs, whereas age, work status, comorbidity, physical and mental status, and use of assistive tools had no influence on the total healthcare costs during rehabilitation and 6 months after the rehabilitation stay. 

## 4. Discussion

This study provides a descriptive analysis of direct and indirect costs incurred by various subgroups of patients with inflammatory or non-inflammatory arthritis receiving multidisciplinary rehabilitation care. Three main findings were observed: first, the total healthcare costs ranged from *€*3,033 to *€*91,336 and were significantly higher at hospital departments compared to rehabilitation centres with mean *€*9,722 (SD 5,406) and *€*4,250 (SD 1,040), respectively. Second, while total costs prior to and after rehabilitation were stable for those receiving rehabilitation at a hospital, there was a significant increase in costs for the three clinical subgroups at a rehabilitation centre. Third, increased total healthcare costs were associated with having rehabilitation in an inpatient hospital department, being a women, using assistive tools, and having reduced physical function in patients with inflammatory arthritis, whereas only female gender influenced healthcare costs among patients with non-inflammatory arthritis. These findings indicate that—for chronic conditions like rheumatic diseases—it is important to look at healthcare consumption and related costs in a wider time perspective than only the rehabilitation period. 

In general, vast cost discrepancies have been reported across studies [17] and across diagnostic subgroups within rheumatic diseases [18]. For example, Flipon et al. [7] found that costs in 2003 attributable to inflammatory arthritis such as rheumatoid arthritis were *€*6,000 and *€*2,400 for undifferentiated arthritis. In a more recent review of cost-of-illness studies from 2007 the yearly cost estimates of rheumatoid arthritis ranged from 11,717 to 28,498 Canadian Dollars (CAD) depending on the method [19]. 

Overall, in-patient hospital costs accounted for the largest proportion of direct healthcare costs, whereas the costs related to primary healthcare and medication accounted for lower costs. Even though the rehabilitation stay at hospitals was significantly shorter than in rehabilitation institutions (16 versus 23 days), the costs for rehabilitation were significantly higher at the hospitals. The main reason for this difference is the large difference in the price per day/night per person in hospitals as compared to rehabilitation centres. This finding is in line with our a priori expectations and also with several previous studies [6, 7, 18] that have reported that in-patient costs represent the largest proportions of healthcare costs. The increase in costs for various subgroups of patients can be defended if there are important reasons to treat patients differently, for example, that a longer and more resource-demanding rehabilitation intervention provides better outcome, and hence are more cost-effective, or that some patients need longer rehabilitation period because they are more seriously affected by the disease. In a previous study [20] we found no significant or clinically important differences in physical or mental function between patients with inflammatory disease receiving rehabilitation in the rheumatology hospital departments and the rehabilitation centres, despite that there were significant differences in the content of team rehabilitation across the clinical settings. Two previous trials, in which multidisciplinary rehabilitation care was compared across different clinical settings, reported a similar finding [3, 4]. Both studies found no significant differences in clinical outcomes, but significantly lower costs in the outpatient clinic as compared to the in-patient program. Moreover, contrary to our expectations, the severity of the inflammatory disease groups was similar across the clinical rehabilitation settings at baseline, thus suggesting that severity cannot explain why some patients were referred to an inpatient hospital stay or a rehabilitation stay. On the other hand, among patients with inflammatory disease we found higher costs for patients with poorer physical function and the need for assistive tools. Such subgroups of patients could be in need for a more resource-demanding and maybe longer rehabilitation period than others. More research needs to identify patient groups which preferably should receive inpatient or outpatient rehabilitation. 

We expected that the healthcare costs prior to a rehabilitation stay were higher as compared to the period after rehabilitation, in particular in the first 3-month period after the rehabilitation stay. An underlying assumption was that patients with arthritis had a reduced need for healthcare in the first months following multidisciplinary rehabilitation care of at least one-week duration. Opposite to our expectations, the healthcare costs were surprisingly high during the whole observation period, in particular for the three clinical subgroups receiving rehabilitation at a rehabilitation centre. Some of the increased costs right after the rehabilitation stay might have been a consequence of interventions that were initiated during the rehabilitation stay followed up in the primary care after discharge. It can also be argued that six months of follow-up was too short time to show that the healthcare costs might have reached the level prior to the rehabilitation stay. On the other hand, similar to the findings in our study a recently published observational study from Germany [6] reported only minor differences in various healthcare costs (hospital, medication, and physical therapy) in the year prior to and the year after an inpatient or outpatient rehabilitation stay. This might indicate that people with rheumatic diseases have a relatively high and constant use of healthcare services in a longer time perspective. Interestingly, healthcare costs in our study are comparable to this German study [6], in particular the mean hospital costs and costs for physical therapy. Even though the studies were performed within different settings with different health care systems, in both Germany and Norway only minor differences exist for “out of the pocket” expenses in rehabilitation settings which in both countries are very low. Costs due to medication use were higher for the patients with inflammatory disease in the present study as compared to the German study, because more of our patients used expensive biological medication. 

The mean direct cost of outpatient rehabilitation in our study was about doubled compared to the German study [6] and was even 5-6 times higher for inpatient hospital rehabilitation. Also compared to another observational study [21], in which a mean cost of inpatient stay with multidisciplinary rehabilitation for patients with RA was calculated to be *€*2,494 (range 915–4,090), the costs estimated in our study were about fourfold. Since comprehensive inpatient and outpatient multidisciplinary rehabilitation program have been found to be equally effective in patients with rheumatoid arthritis, outpatient programmes are recommended [3–5]. Both this study and previous findings from the observational study from Germany [6] suggest that there are similar outcome of inpatient and outpatient rehabilitation and hence support this recommendation. 

The costs due to production loss associated with arthritis exceeded by far the direct costs of healthcare. Although this finding is similar to previous reviews of cost-of-illness studies for patients with arthritis [9, 19], where indirect costs are assumed to represent 50–75% of total costs, our results should be interpreted with caution since only a minority of the patients in our study were eligible for working.

Another limitation is the lower number of patients in the outpatient group, which must be considered when interpreting the results within this group. As mentioned above, one might argue that 6-month follow-up was short and a longer follow-up period could have helped to explore whether costs due to healthcare consumption in the primary care decreased back to the level prior to a rehabilitation stay. Furthermore, the fact that the response rate at 6 months of 66% and nonresponders were slightly younger and more frequently single, with lower level of education and poorer mental health, might indicate a potential for selection bias. High rates of nonrespondents among younger people have also been reported in other studies using mailed surveys [22], and it is not clear whether any of these factors influence cost estimates. Although we had no intention or possibility to draw any conclusions regarding cost-effectiveness in this study, the lack of a control group must also be considered as a limitation. 

The main strengths of this study were a large sample size, further data for all study institutions were concurrently collected, and investigation of the use of healthcare as well as production loss after the rehabilitation stay was performed in a detailed way. We also consider that this study has implications for further research. The observed large differences in costs across clinical settings in which rehabilitation is provided should be tested out in a randomised, controlled trial in order to assess whether the increased costs can be justified with increased effectiveness. Moreover, it is important to investigate whether a good follow-up procedure in the primary care can prevent or reduce the need for costly inpatient multidisciplinary rehabilitation. Finally, the study results can be used in a discussion regarding what is optimal level of healthcare consumption and/or rehabilitation in various subgroups of patients with arthritis. The patients in this study had a lower level of healthcare consumption prior to the rehabilitation period than afterwards. That might seem contradictory as one would assume that their condition was more severely affected before than after. It is crucial to understand the relationship between need and/or use of healthcare consumption and severity of chronic diseases, in order to develop longer-term optimal disease management plans.

To conclude, our data demonstrated substantial costs related to multidisciplinary rehabilitation care and use of healthcare consumption among patients with arthritis, before, during, and after the rehabilitation stay. The time period following a scheduled rehabilitation stay was characterized by surprisingly high healthcare consumption and costs in patients with arthritis. Both the clinical setting in which rehabilitation was provided and disease severity itself had a large impact on healthcare consumption and costs. In patients still working, indirect costs in terms of sick leave were considerable. As a clinical implication, methods to reduce the length of rehabilitation stay should be developed, in particular at hospitals, and regarding production loss with a focus on cost saving, given that health outcomes are constant. Clinical studies on different rehabilitation strategies should include health-economic analyses contributing to the best management of rheumatic diseases.

## Figures and Tables

**Table 1:** Cost categories, units, valuation, and unit price, all numbers in EURO (*€*).

Cost categories	Unit	Valuation	Unit price *€¹*
*Primary care (cost group 1)*			
General practitioner	Visits	NAV	38
Specialist (several types)	Visits	NAV	171
Physiotherapy²	Per treatment	Charge	38
Manual therapy²	Per treatment	Charge	41
Chiropractor²	Per treatment	Charge	63
Occupational therapy²	Per treatment	Charge	41
Others (psychologist, social worker, nurse, practical assistant other types)²	Per treatment	Charge	38
Alternative treatment^3^	Visits	Out of pocket	From 29 to 1,625

*Hospitalizations/inpatient stays (cost group 2)*			
Rehabilitation centre	Days	Direct communication	188
Hospital inpatient unit	Days	DRG 462 B	711
Hospital outpatient unit	Days	NAV	195
Hip/knee prosthesis surgery	Per patient	DRG 211	17,783
Inpatient hospital stay before/after rehab stay	Days	DRG-average	1,193

*Medication (cost group 3)*			
Abatacept (Orencia)	3 set à 250 mg	Price per month	511
Etnaercept (Enbrel)	4 sett (50 mg)	Price per month	665
Adalmimumab, ikke adalmimumab (Humira)	40 mg ∗ 2	Price per month	1,312
Infliximab (Remicade)	3 mg per kilo (300 mg) ∗ 0.5	Price per month	2,201
Tocilizumab (Ro-Actemra)	8 mg per kilo (400 mg) ∗ 2	Price per month	1,246
Anakinra (Kineret)	100 mg per day ∗ 30	Price per month	1,016
Leflunomide (Arava)	20 mg 30 tablets	Price per month	83
Other medication		Price per month	25

*Production loss (sick leave) (cost group 4)*			
	Hours	Wage rate per day	244

*¹*1*€* = 8 NOK.

²Treatment time varies from 30 to 45 minutes.

^3^Treatment modalities such as acupuncture, homeopathy, Thai massage, and Turkey bath/spa rehab stay.

NAV: Norwegian Labor and Welfare Administration.

DRG: diagnosis-related groups.

**Table 2:** Sociodemographic and health variables at baseline for the total sample (*n* = 306) and for each of the diagnostic subgroups at hospital departments (*n* = 62) and rehabilitation centres (*n* = 244).

	ALL *N* = 306	Inflamm. disease hospital (*n* = 48)	Inflamm. disease outpatient hospital (*n* = 14)	Inflamm. disease rehab centre (*n* = 50)	OA conventional rehab centre (*n* = 63)	OA postsurgery rehab (*n* = 131)
Age (y), mean (SD)	60.7 (9.6)	54.4 (11.2)	51.6 (11.2)	58.2 (9.5)	60.7 (8.1)	64.9 (7.1)
Female, *n* (%)	229 (74.8)	26 (54.2)	10 (71.4)	48 (96.0)	61 (96.8)	84 (64.1)
Married/cohabitant, *n* (%)	213 (69.6)	35 (74.5)	11 (78.5)	32 (66.7)	38 (60.3)	97 (75.8)

Education, *n* (%)						
≤9 years	77 (25.2)	15 (31.9)	5 (35.7)	13 (26.5)	15 (24.2)	29 (22.8)
≤12 years	70 (22.9)	11 (23.4)	3 (21.4)	11 (22.4)	18 (29.0)	27 (21.3)
>12 years	152 (49.7)	21 (44.7)	6 (42.9)	25 (51.0)	29 (46.8)	71 (55.9)
Missing	7	1	—	1	1	4

Work situation, *n* (%)						
Employed	40 (13.2)	10 (21.3)	7 (50.0)	6 (12.2)	8 (12.7)	9 (7.0)
Employed, but on sick leave	50 (16.3)	4 (8.5)	4 (28.6)	4 (8.2)	10 (15.9)	28 (21.7)
Disability pension	111 (36.3)	23 (48.9)	2 (14.3)	29 (59.2)	29 (46.0)	25 (19.4)
Age pension	99 (32.4)	7 (14.9)	1 (7.1)	9 (18.4)	16 (25.4)	65 (50.4)
Homeworker	4 (1.3)	2 (4.2)	0	0	0	2 (1.6)
Unemployed	2 (0.7)	1 (2.1)	0	1 (2.0)	0	0
Missing	4	1	—	1	—	2

Co-morbidity, *n* (%)						
None	52 (17.0)	14 (29.2)	5 (35.7)	4 (8.0)	5 (7.9)	24 (18.3)
One	106 (34.6)	17 (35.4)	4 (28.6)	19 (38.0)	11 (17.5)	55 (42.0)
Two or more	148 (48.4)	17 (35.4)	5 (35.7)	27 (54.0)	47 (74.6)	52 (39.7)

Daily use pain medication, *n* (%)	152 (49.7)	23 (53.5)	15 (35.7)	23 (53.5)	3 (30.0)	20 (31.7)
Daily use anti-inflammatory medication, *n* (%)	93 (30.4)	24 (58.5)	6 (54.5)	17 (44.7)	36 (45.0)	93 (43.3)
Daily use relaxing/sleeping med, *n* (%)	51 (16.7)	5 (13.5)	2 (22.2)	12 (30.8)	12 (21.8)	20 (23.0)
Assistive tools, mean (SD)	0.9 (1.8)	1.2 (2.0)	1.9 (2.8)	0.8 (1.7)	0.5 (1.4)	1.0 (1.7)
Days of rehab stay mean (SD)	21.3 (6.2)	16.7 (5.8)	11.9 (2.0)	26.7 (5.9)	25.5 (4.8)	19.8 (3.8)
MHAQ^1^, mean (SD)	.77 (.48)	.71 (.46)	.49 (.43)	.63 (.46)	.65 (.45)	.94 (.46)
SF36 physical sum score^2^, mean (SD)	30.5 (8.9)	34.1 (10.8)	38.3 (8.5)	31.4 (8.1)	30.5 (9.0)	27.8 (7.5)
SF36 mental sum scores^2^, mean (SD)	49.1 (12.7)	47.7 (13.1)	42.6 (13.2)	46.1 (12.3)	48.3 (12.8)	52.0 (12.2)

OA: osteoarthritis; rehab: rehabilitation.

^1^MHAQ score 0–3, 0 = best possible score, 3 = worst possible score.

^2^SF-36 sum scores, score 0–100, 0 = worst possible score, 100 = best possible score.

^3^ *P*-values refer to ANOVA analysis for continuous variables and Chi-square for categorical variable.

**Table 3:** Mean costs in Euro (*€*) with 95% Confidence Intervals_B_ 3 months prior to, during, and 6 months after multidisciplinary rehabilitation in various clinical subgroups; the costs are presented according to the four cost groups.

	Cost group	Pre rehab stay	Rehabilitation stay	0–3 months post rehab stay	4–6 months post rehab stay	*P*-value^2^
Inflamm. disease hospital (*n* = 48)	1	378 (88–422)	—	545 (87–595)	586 (110–649)	.16
	2	2,010 (−1,258–2,776)	11,884 (10,940–13,443)	3,808 (961–7,334)	1,073 (−1,475–1521)	.61
	3	640 (−172–850)	544 (261–587)	1,136 (190–1492)	1,136 (190–1492)	.45*
	4^1^	8,191 (3,209–9,039)	3,158 (2,559–3,296)	8,480 (3,721–9,335)	8,480 (3,721–9,335)	.59*

Inflamm. disease outpatient hospital (*n* = 14)	1	518 (248–645)	—	652 (164–731)	600 (69–715)	.54
	2	1,448 (−1,991–2,385)	2,309 (1,628–2,374)	851 (−1,347–2,172)	85 (−2,035–104)	.40
	3	636 (−786–1,458)	371 (62–440)	637 (−813–1498)	637 (−813–1498)	1.000*
	4^1^	6,059 (−532–8,516)	2,437 (1,891–2,522)	7,583 (1,410–9,849)	7,583 (1,410–9,849)	.20*

Inflamm. disease rehab centre (*n* = 50)	1	483 (362–606)	—	830 (626–1,035)	802 (595–1,009)	.01
	2	1,411 (160–2,663)	5,010 (4,703–5,316)	522 (83–961)	814 (−236–1,865)	.74
	3	232 (−69–534)	665 (545–786)	231 (−64–526)	231 (−64–526)	.76*
	4^1^	9,637 (7,571–11,704)	4,773 (4,451–5,004)	10,215 (8,263–12,167)	10,215 (8,263–12,167)	.77*

OA conventional rehab centre (*n* = 63)	1	479 (181–533)	—	897 (302–1,074)	990 (201–1,367)	.005
	2	2,629 (−691–3,447)	4,780 (4,098–4,855)	165 (−767–238)	1,070 (−1,410–714)	.01
	3	73 (−530–72)	587 (336–600)	72 (−518–71)	72 (−518–71)	.65*
	4^1^	7,997 (3,058–8,703)	4,608 (4,074–4,679)	9,191 (4,589–9,888)	9,191 (4,589–9,888)	.24*

OA postsurgery rehab (*n* = 131)	1	415 (138–448)	—	1,009 (498–1,112)	659 (225–678)	<.001
	2	17,268 (17,142–19,899)	3,704 (3,065–3,733)	1,207 (−67–1,603)	579 (−1,656–714)	<.001
	3	99 (−542–108)	430 (187–433)	89 (−505–91)	89 (−505–91)	.002*
	4^1^	4,863 (438–5,176)	3,636 (3,161–3,648)	6,153 (1,854–6,549)	6,153 (1,854–6,549)	.21*

Cost groups 1–4 refer to costs related to health consumption in the primary healthcare services (cost group 1), secondary healthcare services (cost group 2), use of medication (cost group 3), and costs related to production loss in terms of sick leave (cost group 4). All costs are presented in Euros: 1*€* = 8 NOK.

OA: osteoarthritis; rehab: rehabilitation

_B_ = Bootstrap 95% confidence interval.

^1^Production loss includes only sick leave for employed patients. Age and disability pension are not included.

^2^ *P*-values refer to testing for differences between the three time periods 3 months prior to, 0 to 3 months, and 4 to 6 months after rehabilitation stay within each of the clinical subgroups and cost group. Rank sum based on “Kruskal-Wallis equality-of-populations rank test” was used.

**Table 4:** Mean (SD) and median (min-max) health care costs (summary of 1, 2, and 3) and health care- and social costs (summary of 1, 2, 3, and 4) for the whole observation period. Costs are in Euro (*€*).

	Inflamm. disease hospital (*n* = 48)	Inflamm. disease outpatient hospital (*n* = 14)	Inflamm. disease rehab centre (*n* = 50)	OA conventional rehab centre (*n* = 63)	OA postsurgery rehab (*n* = 131)
Cost group 1, 2 and 3 including rehab stay					
Mean (SD)	23,605 (17,399)	8,749 (7,971)	11,038 (7,998)	11,817 (8,978)	25,136 (7,298)
Median (min-max)	15,145 (7,779–91,336)	5,208 (3,033–27,687)	8,575 (3,900–38,793)	8,019 (5,006–46,703)	24,239 (4,539–48,468)

Cost group 1, 2, 3 and 4 including rehab stay					
Mean (SD)	51,492 (29,894)	32,410 (24,498)	45,880 (24,109)	42,804 (24,288)	45,952 (20,889)
Median (min-max)	59,986 (32,410–24,498)	24,993 (5,714–73,993)	57,801 (8,040–92,906)	52,065 (9,196–89,801)	40,993 (7,951–90,491)

Cost groups 1–4 refer to costs related to health consumption in the primary healthcare services (cost group 1), secondary healthcare services (cost group 2), use of medication (cost group 3), and costs related to production loss in terms of sick leave (cost group 4). All costs are presented in Euros: 1*€* = 8 NOK.

OA: osteoarthritis; rehab: rehabilitation.

*P*-values refer to Kruskal Wallis testing for differences within period and cost group, but between diagnostic and clinical setting.

**Table:** (a) The influence of clinical subgroup, age, gender, comorbidity, and work status on total health care costs (sum of cost groups 1 to 3 for rehabilitation period and 6 months after) for patients with inflammatory disease. The results are presented as Unstandardised Coefficients (*β*) with 95% Confidence intervals

Variable	Model 1	Model 2	Model 3	Model 4
Clinical subgroup				

*Inflammatory disease rehab center*	Ref.	Ref.	Ref.	Ref.

Inflammatory disease inpatient hospital unit	13,412 (8,744 to 18,080)**	12,952 (8,299 to 17,605)**	12,935 (8,173 to 17697)**	13,721 (8,806 to 18,635)**

Inflammatory disease outpatient hospital unit	−2,595 (−9,388 to 4,196)	−2,406 (−5,875 to 3,727)	−2,560 (−9,514 to 4,395)	−73 (−7,089 to 6,940)

Gender *(ref. female)* Male	−2,968 (−8,055 to 2,118)	−3,272 (−8,371 to 1,826)	−3,555 (−8,744 to 1,633)*	−4,025 (−9,402 to 1,352)

Age *(ref. 67 or less)* 68+	1,682 (−4,712 to 8,077)	1,843 (−4,583 to 8,271)	4,430 (−1,762 to 10,624)	4,045 (−2,379 to 10,469)

Comorbidity* (ref. none) *				
One	3,170 (−2,373 to 8,714)	3,291 (−2,282 to 8,864)	3,795 (−1,849 to 9,439)	4,851 (−1,002 to 10,632)
Two or more	2,939 (−2,830 to 8,708)	2,274 (−3,457 to 8,005)	2,885 (−3,003 to 8,775)	3,346 (−2,759 to 9,452)

Working status* (ref. employed)* Not employed	1,732 (−3,288 to 6,753)	1,716 (−3,333 to 6,765)	448 (−4,589 to 5,485)	402 (−4,827 to 5,632)

MHAQ	−5,179 (−12,174 to 1,815)	—	1,151 (−4,092 to 6,396)	4,122 (−921 to 9,166)*

SF Physical function	−172 (−301 to −42)**	−106 (−202 to −11)*	—	—

SF Mental function	−1 (−21to 19)	41 (−21 to 19)	—	—

Assistive tools	1,447 (414 to 2,540)**	1,315 (268 to 2,361)*	1,619 (542 to 2,695)**	—

Constant	16,367 (4,568 to 28,168)**	10,292 (10763 to 18,819)**	3,367 (−3,134 to 9,869)	2,178 (−4,522 to 8,878)

Adj R-squared	0.3593	0.3518	0.3276	0.2750

Cost values (in Euro) are estimated means by OLS. All costs are presented in Euros: 1*€* = 8 NOK.

*P-value <  .05, **P-value <  .01.

The difference between the three models is only the inclusion of different severity measures in addition to clinical setting, diagnosis, age, gender, and working status.

Model 1: all severity measures are included: MHAQ, SF36 Physical function, SF36 Mental function, and assistive tools.

Model 2: we include only SF36 Physical function, SF36 Mental function and assistive tools.

Model 3: we include only MHAQ and assistive tools.

Model 4: we include only MHAQ.

**Table:** (b) The influence of clinical subgroup, age, gender, comorbidity, and work status on total health care costs (sum of cost groups 1 to 3 for rehabilitation period and 6 months after) for patients with osteoarthritis (OA). The results are presented as Unstandardised Coefficients (*β*) with 95% Confidence intervals

Variable	Model 1	Model 2	Model 3	Model 4
Clinical subgroup				

*OA (ref. conventional rehabilitation)*	Ref.	Ref.	Ref.	Ref.

OA postsurgery rehabilitation	−675 (−2,664 to 1,313)	−220 (−2,135 to 1,694)	−683 (−2,658 to 1,290)	−57 (−2,723 to1,207)

Gender *(ref. female)* Male	−2,086 (−4,166 to −6)*	−2,037 (−4,125 to −50)	−2,103 (−4,142 to −65)*	1,915 (−3,903 to 72)

Age *(ref. 67 or less)* 68+	651 (−1,282 to 2585)	386 (−1,527 to 2,300)	693 (−1,217 to 2,605)	578 (−1,312 to 2,468)

Comorbidity *(ref. none) *				
One	1,054 (−1,445 to 3,554)	1,080 (−1,429 to 3,590)	1,178 (−1,265 to 3,622)	1,148 (−1,292 to 3,589)
Two or more	1,341 (−1,160 to 3,842)	1,470 (−1,036 to 3,977)	1,514 (−891 to 3,920)	1,507 (−335 to 3,163)

Working status* (ref. employed)* Not employed	192 (−1,805 to 2,190)	−34 (−2,020 to 1,951)	263 (−1,707 to 2,234)	128 (−1,814 to 2,072)

MHAQ	1,512 (−365 to 3,390)	—	1,614 (−199 to 3,428)	1,414 (−335 to 3,163)

SF Physical function	−2 (−13 to 9)	−4 (−15 to 6)	—	—

SF Mental function	−1 (−9 to 6)	−2 (−9 to 6)	—	—

Assistive tools	−216 (−738 to 305)	−106 (−612 to 399)	−220 (−739 to 299)	—
Constant	6,592 (3,301 to 9,882)**	7,650 (4,621 to 10,679)**	6,158 (3,303 to 9,013)**	6,244 (3,399 to 9,090)**

Adj R-squared	0.0101	0.0128	0.0193	0.0209

Cost values (in Euro) are estimated means by OLS. All costs are presented in Euros: 1*€* = 8 NOK.

*P-value <  .05, **  *P*-value <  .01.

The difference between the three models is only the inclusion of different severity measures in addition to clinical setting, diagnosis, age, gender and working status.

Model 1: all severity measures are included: MHAQ, SF36 Physical function, SF36 Mental function, and assistive tools.

Model 2: we include only SF36 Physical function, SF36 Mental function, and assistive tools.

Model 3: we include only MHAQ and assistive tools.

Model 4: we include only MHAQ.
